# Outcome of Breast Cancer in Moroccan Young Women Correlated to Clinic-Pathological Features, Risk Factors and Treatment: A Comparative Study of 716 Cases in a Single Institution

**DOI:** 10.1371/journal.pone.0164841

**Published:** 2016-10-19

**Authors:** Meriem Slaoui, Fatima Zahra Mouh, Imane Ghanname, Rachid Razine, Mohammed El Mzibri, Mariam Amrani

**Affiliations:** 1 Equipe de recherche ONCOGYMA, University of Mohamed V Rabat, Faculty of Medicine and Pharmacy of Rabat, Avenue Mohammed Belarbi El Alaoui–Souissi–BP 6203 Rabat, Morocco; 2 Unité de Biologie et Recherche Médicale. Centre National de l'Energie, des Sciences et des Techniques Nucléaires, Rabat, Morocco; 3 Faculty of Medicine and Pharmacy, University Mohammed V Rabat, Avenue Mohammed Belarbi El Alaoui–Souissi–BP 6203 Rabat, Morocco; 4 Laboratory of Biostatistics, Epidemiology and Clinical Research, University of Mohamed V Rabat, Faculty of Medicine and Pharmacy of Rabat, Avenue Mohammed Belarbi El Alaoui–Souissi–BP 6203 Rabat, Morocco; 5 Department of Public Health, University of Mohamed V Rabat, Faculty of Medicine and Pharmacy of Rabat, Avenue Mohammed Belarbi El Alaoui–Souissi–BP 6203 Rabat, Morocco; Cleveland Clinic Lerner Research Institute, UNITED STATES

## Abstract

**Background:**

Breast cancer in young women is quite uncommon and shows more aggressive characteristics with major disparities between worldwide populations. Prognosis and outcome of breast cancer in young patients are widely studied, but still no consensus is available.

**Methods:**

We retrospectively included 716 cases of breast cancer women diagnosed in 2009 at the National Institute of Oncology of Rabat. Patients were divided into two groups according to their age: women aged ≤40 years (Group 1) and women aged >40 years (Group 2). Data were recorded from patients’ medical files and analyzed using SPSS 13.0 software (IBM).

**Results:**

Young patients represent 24.9% of all patients with breast cancer. The comparison between the two groups displayed significant differences regarding nulliparity (*p* = 0.001) and progesterone receptor negativity (*p* = 0.01). Moreover, more progression (Metastases/Relapse) was registered in young women as compared to older women with breast cancer (*p* = 0.03).

The estimated median follow-up period was 31 months. The 5-years Event-Free Survival (EFS) of patients with local disease was 64.6% in young women and 71.5% in older women with breast cancer (*p* = 0.04). Multivariate analysis in young women showed that nulliparity (HR: 7.2; 95%CI: 1.16–44.54; *p* = 0.03), T3 tumors (HR: 17.39; 95%CI: 1.74–173.34; *p* = 0.01) and negative PgR status (HR: 19.85; 95%CI: 1.07–366.54; *p* = 0.04) can be considered as risk factors for poorer event free survival while hormone therapy was associated with better EFS (HR: 0.11; 95%CI: 0.00–0.75; *p* = 0.03). In Group 2, multivariate analysis showed that patients with inflammatory breast cancer, N+ status, absence of radiotherapy, absence of chemotherapy, and absence of hormone therapy are at increased risk of recurrence.

**Conclusions:**

In Morocco, breast cancer is more frequent in young women as compared to western countries. Breast cancer in young women is more aggressive and is diagnosed late, leading to an intensive treatment. Moreover, the main factors associated with breast cancer development in young women would be hormonal and reproductive status. Analysis of other genetic biomarkers is needed to explain the high prevalence of breast cancer in young women to improve breast cancer management in Morocco.

## Introduction

Worldwide, breast cancer (BC) is the first leading cancer in women with nearly half million deaths annually [1]. Breast cancer in young women is uncommon and very aggressive [2]. In the literature, there is not a wide definition of young women with breast cancer; sometimes they are defined as women under 35, sometimes women under 40 or 50 years [3–5]. In other publications, young women are attributed to all premenopausal women [6].

In developed countries, approximately 5–7% of breast cancer patients are diagnosed before 40 years [7], while in developing countries, the prevalence is much higher. In Morocco, the prevalence of Breast cancer in young women vary between 8% and 25.4%, which represent the highest levels published so far [3, 8, 9].

According to many authors, it has specific epidemiological, diagnostic and prognostic characteristics; up to consider youth as a pejorative prognostic factor [10]. In young patients, histological grade is usually high, the expression of hormone receptors is less important while overexpression of HER2 (Human epidermal growth factor receptor 2) is higher than in older patients. In this subgroup, the triple negative tumors are more common [2]. The occurrence of cancer in this age generates fertility and sexuality problems, mainly related to aggressive treatments [11].

The identification of risk factors related to this disease and the optimization of care pathways are essential to optimize the cancer management, to enhance the chance of complete healing and to improve the life quality of patients.

Therefore, this retrospective study was planned to characterize breast cancer in young women as compared to breast cancer in older women. Characterization of Breast cancer in young women will focus on the epidemiological, clinic-pathological, biomarker expression and treatment characteristics. The comparison will be also applied on the recurrence and survival to identify the prognostic factors. Additionally, other risk factors, including oral contraceptive use, family history of breast cancer and obesity, will be assessed for their association with the aggressive development of breast cancer in this subgroup.

## Methods

### Study design and population

Our study consists of all breast cancer in Moroccan women diagnosed and/or followed up at the National Institute of Oncology in Rabat, Morocco during 2009. A total of 905 patients were recorded. Men patients, cases with missed data and foreign patients (n = 189) were then excluded. The remaining 716 breast cancer cases were divided according to their age into two groups. Group 1 (G1), regrouping 178 patients aged 40 years or less, and Group 2 (G2) including 538 patients more than 40 years old. The median age of young women at diagnosis was 36.5 ± 4.11, for older women, it was 52 ± 9.6.

### Data collection

Data were obtained from patients’ medical files. The medical records were retrospectively reviewed and collected using SPSS-software. For each case, we abstracted all information on age, parity, weight/height, and hormonal status, familial history of breast cancer, clinical data, cancer stage, tumor size, histological type, tumor grade, lymph node involvement, metastases, hormonal receptors, treatment (surgery, chemotherapy, radiation therapy, hormonal therapy) and follow-up.

Histological type was updated according to the WHO classification of breast tumors 2012 (World Health Organization) [12]. Tumor pTNM (pathological Tumor Node Metastasis) staging is consistent with the seventh edition of AJCC classification (American Joint Committee on Cancer) of 2009. Tumor grade was assessed according to Scarff-Bloom & Richardson (SBR) grading system modified by Ellis and Elston [13] and vascular invasion was quantified histologically.

Estrogen and Progesterone receptors (ER and PR) were considered positive when nuclear expression was observed in at least 10% of the tumor cells.

Immuno-histo-chemical expression of Her 2 was defined according to cytoplasmic membrane staining of the infiltrative component taking into account the complete or incomplete membrane staining, the intensity and the % of cells stained. Results are expressed in scores; 0/1+: negative, 2+: ambiguous and 3+: positive. In cases of ambiguous, Fluorescent *in situ* hybridization (FISH) was performed to assess Her 2 amplification. If Her 2 amplification is confirmed by FISH, the result was considered as positive.

According to ER, PR and Her2 status, breast cancer cases were classified into five subgroups: Luminal A (ER+/PR+/Her2-), Luminal B Her2- (ER+/PR- or lower than 20% /Her2-), Luminal B Her+ (ER+/PR+ or—/Her2+), Her2 (ER-/PR-/Her2+) and triple negative (ER-/PR-/Her2-) [14].

### Follow-up

Patients were followed up until December 2014. Event free survival (EFS) was calculated from the date of surgery or the date of starting chemotherapy to the date of loco-regional recurrence or distant metastasis.

### Statistical analysis

Statistical analysis was assessed by SPSS 13.0 software (IBM). Descriptive variables were expressed as means ± SD or medians (interquartile range). The χ2 test was used to analyze differences between qualitative data. Calculation of survival rates was performed by the Kaplan-Meier method and compared using the Log-rank test.

Univariate and multivariate Cox’s regression model was performed to compare variables and outcome. A value of p < 0.05 is considered significant. In the multivariate model, all parameters reported in previous studies as influencing survival rates were included. These parameters were not necessarily significant in the univariate model.

### Ethics approval

The study was approved by the Ethical Committee of Biological Research, Faculty of Medicine and Pharmacy–Rabat, and was conducted with respect to legal aspects. No consent was needed for this retrospective study and data were re-identified.

## Results

### Clinicopathological characteristics

Clinical and pathological data are reported respectively in Tables 1 and 2. Overall, breast cancer in Morocco is characterized by a median age at diagnosis of 48.9 ± 11.6, with extreme ages at 21 and 89 years. BC cases are mainly sporadic and only 14.5% of cases have a familial history of BC, with low level of metastatic evolution (only 18.2%) and presents a predominance of median stages (69.6% of cases have BC with stages II and III). In young women, clinical data report the same distribution as reported for breast cancer cases. Comparison between young and older women showed a statistically significant difference for metastatic progression (*p* = 0.03). Indeed, 26.9% of young patients exhibited metastatic/relapse progression compared to 17.3% of women over 40 years old. For the other parameters, no statistically significant difference was observed. Interestingly, only 10.02% of young women with BC are obese, whereas in older women 33.6% of cases are obese.

**Table 1:** Comparative clinical data by age groups.

Variables		All patients (%)	Number of patients ≤ 40y (%)	Number of patients > 40y (%)	*p-*value
Nulliparity	Yes	160 (24.6)	59 (35.3)	101 (21.0)	**0.001**
	No	489 (75.4)	108 (64.7)	381 (79.0)	
Number of full term pregnancies	0	150 (23.4)	56 (33.9)	94 (19.7)	**0.001**
	2	58 (9.0)	19 (11.5)	39 (8.2)	
	2–4	226 (35.3)	73 (44.2)	153 (32.1)	
	≥5	207 (32.3)	17 (10.3)	190 (39.9)	
Oral contraceptives use	Yes	188 (40.0)	62 (46.3)	126 (37.5)	0.08
	No	282 (60.0)	72 (53.7)	210 (62.5)	
Menopausal status	Pre-menopausal	282 (44.5)	0 (0.0)	282 (59.2)	**0.001**
	post-menopausal	351 (55.5)	157 (100)	194 (40.8)	
Familial history of breast cancer	Yes	83 (14.6)	22 (15.2)	61 (14.4)	0.80
	No	487 (85.4)	123 (84.8)	364 (85.6)	
Obesity	Yes	112 (27.9)	10 (10.2)	102 (33.6)	**0.001**
	No	290 (72.1)	88 (89.8)	202 (66.4)	
Metastatic disease	Yes	117 (18.2)	23 (13.8)	94 (19.7)	0.10
	No	527 (81.8)	144 (86.2)	383 (80.3)	
Progression (Metastasis/relapse)	Yes	79 (19.9)	29 (26.9)	50 (17.3)	**0.03**
	No	318 (80.1)	79 (73.1)	239 (82.7)	
stage	I	56 (9.8)	16 (11.5)	40 (9.3)	0.32
	II	237 (41.6)	55 (39.6)	182 (42.3)	
	III	159 (28.0)	45 (32.4)	114 (26.5)	
	IV	117 (20.6)	23 (16.5)	94 (21.9)	

**Table 2:** Comparative pathological data by age groups.

Variables		All patients (%)	Number of patients ≤ 40y (%)	Number of patients > 40y (%)	*p-*value
ER	positive	412 (68.1)	113 (73.4)	299 (66.3)	0.10
	negative	193 (31.9)	41 (26.6)	152 (33.7)	
PgR	positive	432 (71.9)	123 (79.9)	309 (69.1)	**0.01**
	negative	169 (28.1)	31 (20.1)	138 (30.9)	
HER2	positive	116 (23.0)	33 (24.8)	83 (22.4)	0.57
	negative	387 (77.0)	100 (75.2)	287 (77.6)	
Molecular subtype	Luminal A	261 (52.2)	74 (55.6)	187 (51.0)	0.27
	Luminal B HER2-	42 (8.4)	6 (4.5)	36 (9.8)	
	Luminal B HER2+	80 (16.0)	25 (18.8)	55 (15.0)	
	HER2	33 (6.6)	7 (5.3)	26 (7.1)	
	Triple negative	84 (16.8)	21 (15.8)	63 (17.2)	
Tumor size	≤20mm	101 (18.0)	28 (21.4)	73 (18.5)	0.43
	21–50 mm	298 (53.2)	68 (51.9)	230 (58.4)	
	>50mm	161 (28.8)	35 (26.7)	126 (23.1)	
Lymph nodes	N0	217 (40.0)	53 (39.0)	164 (40.3)	0.51
	N1	176 (32.4)	41 (30.1)	135 (33.2)	
	N2	89 (16.4)	22 (16.2)	67 (16.5)	
	N3	61 (11.2)	20 (14.7)	41 (10.1)	
Histological type	Invasive carcinoma of NST	567 (85.4)	142 (86.6)	425 (85.0)	0.08
	Invasive lobular carcinoma	26 (4.0)	10 (6.1)	16 (3.2)	
	Others	71 (10.6)	12 (7.3)	59 (11.8)	
Vascular invasion	Yes	228 (38.1)	60 (40.8)	168 (37.2)	0.42
	No	371 (61.9)	87 (59.2)	284 (62.8)	
SBR grade	SBR I	46 (7.5)	9 (5.9)	37 (8.1)	0.08
	SBR II	373 (61.1)	85 (55.6)	288 (62.9)	
	SBR III	192 (31.4)	59 (38.6)	133 (29.0)	

Her2: Human Epidermal Receptor-2; ER: Estrogen Receptor; PgR: Progesterone Receptor; N: Nodes; NST: No Special Type; SBR: Scarff-Bloom Richardson classification.

Pathological data showed that BC is mainly ER+ (68.1%), PR+ (71.9%) and HER2- (77%). In our cohort, 16.8% of cases are triple negative, 40% have lymph node N0. Histological data showed that invasive carcinoma and the SBR grade II prevail. In young women, pathological data report the same distribution as reported for breast cancer cases. Comparison between young and older women showed a statistically significant difference for progesterone receptor expression (PgR). In fact, 79.9% of young women with BC are PgR+, whereas PgR positivity was reported only in 69.1% of older women with BC (*p* = 0.01). For the other parameters, no statistically significant difference was observed. Of particular interest, 38.6% of young patients and only 29% of older women have SBR grade III. Even statistical analysis showed that there is no difference, a tendency to high SBR grade is observed for young women.

### Treatment

Overall, young and older women received the same treatments. Neoadjuvant chemotherapy was given to 26 young women (18.3%) and to 76 older women (18.1%) (*p* = 0.90). Radical mastectomy along was undergone to 74.9% of older women and to 70.8% of young women (*p* = 0.30). Adjuvant chemotherapy was administered to 73.5% of young women and to 69.1% of older women (*p* = 0.30).

Herceptin treatment, reserved to patients Her2 positives, was given to 11.6% of young patients (19/164) and to 7.3% of older women (34/468). Statistical analysis showed that there is no significant difference using this targeted therapy between young and older women (*p* = 0.08).

Hormonal therapy was provided to patients with positive hormone receptors. Overall, 53.7% of young patients (88/164) and 48.6% of older patients (228/469) have received hormonal therapy, with no significant difference (*p* = 0.26). Finally, radiotherapy was performed on nearly 57% of patients from each group with no statistical difference (*p* = 0.90).

### Data analysis of metastatic patients

Among the 716 subjects of our database, 117 (16.34%) patients were metastatic at diagnosis. Among them, 23 are young women and 94 are older women, representing respectively 13.8% and 19.7% of all young and older women with breast cancer. Table 3 illustrates the comparison of some relevant parameters in the two groups of patients with metastatic disease.

**Table 3:** Characteristics of patients with metastatic disease.

Variables		Metastatic disease		
		women ≤ 40y (%)	women > 40y (%)	*p-*value
Nulliparity	Yes	7 (33.3)	66 (78.6)	0.25
	No	14 (66.7)	18 (21.4)	
Oral contraceptives use	Yes	8 (53.3)	12 (24.0)	**0.03**
	No	7 (46.7)	38 (76.0)	
ER	Positive	15 (75.0)	57 (77.0)	0.53
	Negative	5 (25.0)	17 (23.0)	
PgR	Positive	19 (95.0)	54 (74.0)	**0.03**
	Negative	1 (5.0)	19 (26.0)	
Mestastasis localization	Bone	6 (26.1)	42 (44.7)	0.21
	Liver	6 (26.1)	23 (24.5)	
	Lungs	4 (17.4)	16 (17.0)	
	Multiple locations	7 (30.4)	13 (13.8)	
Obesity	Yes	0 (0.0)	22 (35.5)	**0.02**
	No	10 (100)	40 (64.5)	

ER: Estrogen receptor; PgR: Progesterone Receptor.

Overall, no statistical difference was observed between young and older patients regarding the nulliparity at breast cancer diagnosis, ER status and metastasis localization. However, older women have the tendency to develop metastasis in bone (44.7%), whereas young patients develop metastasis in many organs, including bone (26.1%) and liver (26.1%).

Oral contraception use and progesterone receptor expression were statistically significant with *p*-values of 0.03 for the two parameters. In fact, 95% of young women (19/20) and only 74% of older women (54/73) have a high expression of PgR. Among young women, 53.3% use oral contraception (8/15) whereas only 24% of older women (12/50) had used it. Interestingly, no young woman is obese and 35.5% of older women (22/62) are obese.

### Event Free Survival (EFS) analysis

The estimated median follow-up period was 31 months [11–53] with a range of 3 to 87 months. During the follow-up period, 29 young patients (26.9%) and 50 older patients (17.3%) had recurrence (*p* = 0.03).

**Fig 1:**
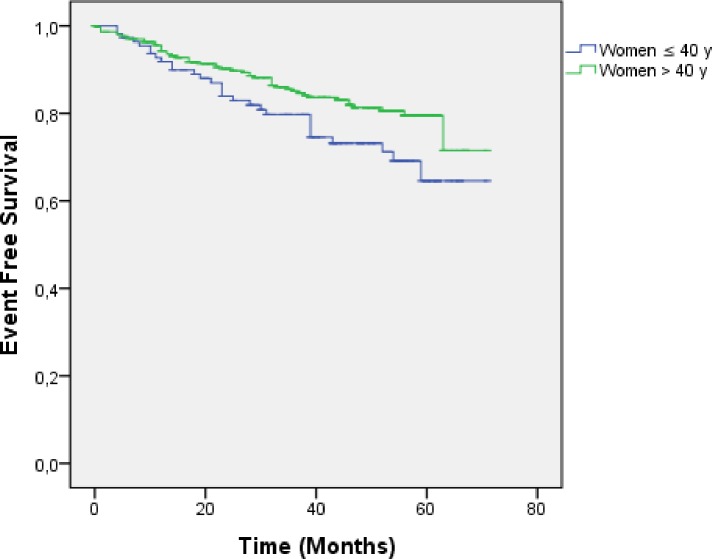
Event free survival (EFS) by age in patients with local disease.

Event free survival (EFS) was calculated using univariate analysis by Kaplan-Meier method. The results are reported in Fig 1. The 3-years EFS of patients with local disease were 74.6% and 85.1% for young and older women, respectively. The 5-years EFS was also higher in older patients (71.5%) than in young patients (64.6%) and this difference is statistically significant (*p* = 0.04).

**Fig 2:**
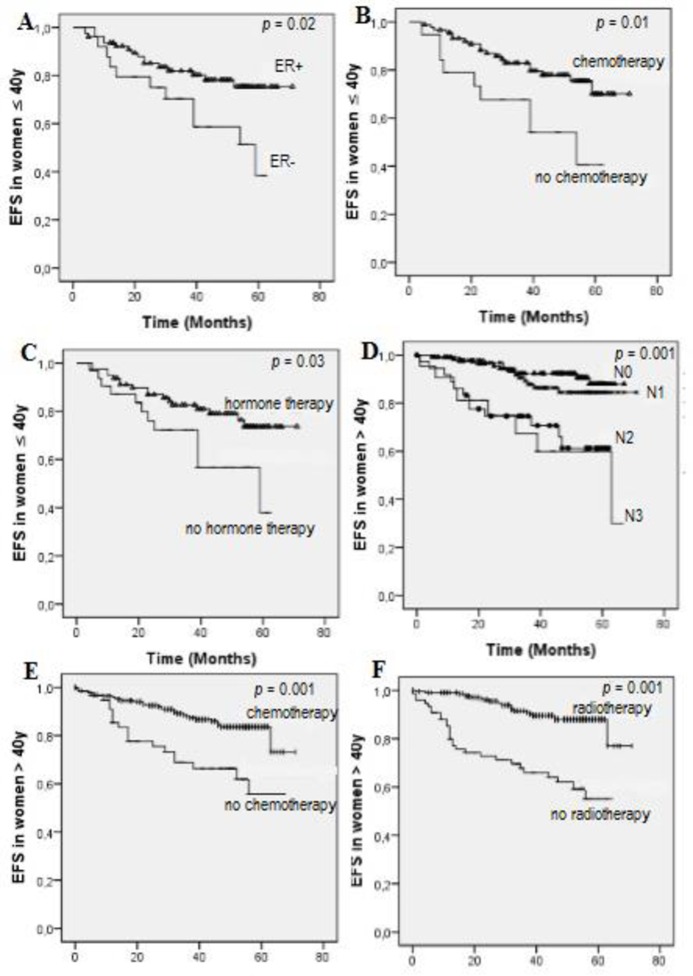
**Event free survival (EFS) correlated to some parameters in young women (A, B, C) and aged women (D, E, F).** (N0/N1/N2/N3: lymphnodes).

Results of EFS correlation to some relevant parameters are represented in Fig 2. EFS is poorer in young women with negative estrogen receptors (*p* = 0.02). In aged women, EFS is better in patients with negative lymphnodes (*p* = 0.00).

### Univariate and Multivariate Cox regression analysis

The results of univariate and multivariate Cox regression analysis are reported in Table 4. Univariate analysis indicated that T3 tumors size, ER negative, absence of chemotherapy and absence of hormonal therapy are statistically the significant parameters influencing event free survival in young women. The multivariate analysis in the same group showed that the absence of hormonal therapy along with negative PgR status, T3 tumors size and nulliparity are associated with poorer EFS.

**Table 4:** Univariate and multivariate Cox regression analysis for Event free survival (EFS).

Parameters		women ≤ 40y						women ≤ 40y					
		Univariate analysis			Multivariate analysis			Univariate analysis			Multivariate analysis		
		HR	95% CI	*p*	HR	95% CI	*p*	HR	95% CI	*p*	HR	95% CI	*p*
Nulliparity	No	1			1			1			1		
	Yes	1.41	0.66–3.02	0.37	7.20	1.16–44.54	**0.03**	0.57	0.24–1.35	0.20	0.30	0.06–1.46	0.13
Oral contraceptives use	No	1						1					
	Yes	0.74	0.32–1.70	0.48				0.57	0.25–1.28	0.17			
Familial history of Breast cancer	No	1						1					
	Yes	1.25	0.42–3.67	0.68				0.57	0.20–1.60	0.28			
Obesity	No	1						1					
	Yes	1.11	0.32–3.81	0.86				1.15	0.58–2.30	0.67			
Inflammatory breast cancer	No	1						1			1		
	Yes	0.64	0.35–1.18	0.15				1.66	1.45–1.96	**0.03**	3.70	1.18–11.58	**0.02**
Tumor size	≤20mm	1			1			1			1		
	21–50 mm	0.88	0.27–2.91	0.84	1.35	0.25–7.29	0.72	2.69	1.00–5.32	**0.049**	0.86	0.18–4.03	0.85
	>50mm	4.94	1.29–18.9	**0.01**	17.39	1.74–173.34	**0.01**	0.58	0.57–5.70	0.31	0.17	0.01–2.41	0.19
SBR grade	SBR I	1						1					
	SBR II	0.79	0.27–2.29	0.67				1.19	0.27–5.09	0.81			
	SBR III	1.15	0.39–3.42	0.79				1.99	0.45–8.63	0.35			
N status	N-	1			1			1			1		
	N+	1.1	0.49–2.42	0.81	3.44	0.55–21.45	0.18	2.48	1.21–5.06	**0.01**	6.70	1.27–35.18	**0.02**
Stage	I	1						1					
	II	2.77	0.35–21.7	0.33				1.19	0.30–3.78	0.88			
	III	3.84	0.49–30.1	0.20				1.21	0.27–3.02	0.87			
ER	positive	1			1			1			1		
	negative	0.38	0.18–0.83	**0.01**	0.41	0.06–2.54	0.34	0.91	0.5–1.64	0.76	1.33	0.24–7.16	0.73
PgR	positive	1			1			1			1		
	negative	0.59	0.26–1.36	0.21	19.85	1.07–366.54	**0.04**	0.86	0.47–1.59	0.64	4.58	0.35–60.10	0.24
HER2	positive	1			1			1			1		
	negative	0.73	0.27–1.96	0.54	6.79	0.62–73.99	0.11	1.48	0.7–3.13	0.29	1.99	0.47–8.43	0.34
Molecular subtype	Luminal A	1						1					
	Luminal B HER2-	3.20	0.9–11.42	0.07				0.54	0.12–2.31	0.40			
	Luminal B HER2+	0.85	0.24–3.05	0.81				1.26	0.50–3.12	0.61			
	HER2	1.29	0.29–5.80	0.73				2.23	0.66–7.52	0.19			
	Triple negative	1.74	0.61–4.95	0.29				1.29	0.55–3.05	0.55			
Surgery type	Radical mastectomy	1			1			1			1		
	Conserving surgery	0.54	0.20–1.44	0.22	3.75	0.61–22.87	0.15	0.76	0.36–1.58	0.46	0.00	0.00—˃˃˃	0.96
Radiotherapy	No	1			1			1			1		
	Yes	0.51	0.23–1.10	0.08	1.15	0.11–11.16	0.90	0.19	0.11–0.34	**0.001**	0.15	0.03–0.71	**0.01**
Chemotherapy	No	1			1			1			1		
	Yes	0.45	0.19–1.00	**0.0049**	1.15	0.11–21.99	0.74	0.37	0.21–0.67	**0.001**	0.12	0.03–0.50	**0.001**
Trastuzumab	No	1			1			1			1		
	Yes	0.42	0.10–1.78	0.24	0.11	0.00–4.15	0.23	0.86	0.31–2.41	0.78	3.14	0.36–26.78	0.29
Hormone therapy	No	1			1			1			1		
	Yes	0.41	0.19–0.86	**0.01**	0.11	0.00–0.75	**0.03**	0.35	0.20–0.61	**0.001**	**0.03**	0.00–0.27	**0.001**

HR: hazard Ratio; CI: confidence interval; SBR: Scarff-Bloom Richardson classification; N: Node.

In the older group, univariate analysis showed that inflammatory breast cancer; T2 tumors, N+ status, absence of radiotherapy, absence of chemotherapy and absence of hormone therapy are associated with poorer EFS. The same results were found with the multivariate analysis except for T2 tumors, which were not associated with poorer EFS.

## Discussion

The prevalence of breast cancer in young women is low but the impact of the disease is significant. In this study, young women under 40 years represent 24.9% of all women with breast cancer. Previous studies in Morocco reported disparate results, ranging from 8 to 25.4 [3, 8, 9, 15]. Worldwide, the prevalence of breast cancer in young women is variable. In USA, breast cancer in young women is lower, only 6.4% of patients with breast cancer are under 40 years [4]. The same data was reported in Italy [16]. However, recent study conducted in Switzerland, patients aged between 20 and 39 years represented 23.4% of all breast cancer cases which concords with our findings [17]. In Algeria, young women represent 12% of breast cancer cases [18].

Breast cancer is a complex and heterogeneous disease associated with clinical, pathological and biological factors largely variable from a population to another. To our knowledge, the current analysis represents the first large comparative study of risk factors as outcome predictors in young versus older breast cancer patients in Morocco. It was conducted in the National Institute of Oncology in Rabat, considered as a reference public health oncology center.

In this study, nulliparity at diagnosis is more frequent in young women with breast cancer than in older women with a significant difference. Our results agree with already reported studies. Indeed, previous studies have focused their interest on the nulliparity status as a risk factor for breast cancer development. MacMahon have reported that nulliparous women have a higher risk of breast cancer than parous women [19]. In a Japanese study, Tamakoshi et al. have clearly showed that reproductive factors, particularly the number of parity and age at first delivery, might be important in the etiology of breast cancer among Japanese women [20]. The increased risk of breast cancer in nulliparous women could be attributed to the high levels of prolactin and circulating oestradiol than in parous and/or older women [21]. Moreover, high parity increases the initiation of tumor cells during breast tissue maturation that occurs repeatedly with every pregnancy [22], explaining the overall high frequency of multiparity in young and older breast cancer women.

Currently, it is widely accepted that obesity increases BC risk in postmenopausal women and is associated with reduced risk of BC for premenauposal women [23]. In our study, only 10.2% of young women and 33.6% of older women with breast cancer were obese. In premenopausal women, obesity is associated with absence of ovulation and lower levels of circulating estrogen levels that decrease the risk of developing breast cancer [23].

Tumors in the young group are more aggressive with high SBR grades, lymph node involvement and high tumor size, but still not significant when compared with the older group. Several studies indicate that tumors in young women are more advanced and explained it by the delay of diagnosis and the lack of awareness [3, 8, 24].

The difference in hormone receptors expression was also investigated and a statistically significant difference was observed for PgR. PgR positivity is usually correlated to a better prognosis and less recurrence [25]. In this study, the expression of PgR in young women was more frequent than in older women with breast cancer (*p* = 0.01). Our results are in agreement with previous reported data showing that postmenopausal women have low PgR expression than premenopausal women [26].

ER status did not show a significant difference between young and older women with breast cancer, while many studies have demonstrated more negativity in hormonal receptors in young women [4, 27]. Of particular interest, ER status is influenced by the patient’s obesity. In fact, obese postmenopausal women are more likely to be ER+ than obese premenopausal women, because adipose tissue is the primary source of estrogen production via aromatase enzyme conversion of androgenic precursors increasing the risk of breast cancer [23, 25].

Currently, a great interest was given to molecular subtypes as factors influencing breast cancer outcome. Accordingly, TNBC are more aggressive and show low clinical and pathological response to chemotherapy compared to the remaining subtypes, especially Luminal A [28]. Many authors showed that TNBC is more frequent in young women [29, 30]. In our study, TNBC was reported in both young and older women with no statistical difference. Moreover, TNBC was associated with poorer EFS, which is in agreement with previously results [28, 29, 31]. Hormonal treatment is always correlated to better outcome hence the interest of this therapy[3]. The non-administration of hormonal therapy to TNBC subtypes, due to their hormones receptors status, may explain the great association between TNBC and poorer EFS. Therefore, and in the absence of genetic profiling, molecular subtypes are still a good prognostic factor of response to adjuvant treatments and survival prediction.

Relapse was more frequent in young women as compared to older women with breast cancer (*p* = 0.03). This difference could be explained by the tumor aggressiveness in this subgroup as reported in many studies [2, 10, 32]. In fact, SBR III grade and high tumor size prevail in young women than older women.

Kaplan-Meier analysis clearly showed that EFS in young women was poorer than in older women, which is in agreement with already published results in Morocco and other countries [3, 8, 33]. It is widely accepted that survival rate can be influenced by several parameters including oral contraception use and nulliparity. In this study, results highlighted that EFS is poorer among oral contraceptive users. Recent studies found an increased risk of breast cancer among OC users [34, 35], but this correlation is still controversial and subject to several ongoing studies.

EFS was also influenced by the nulliparity status at breast cancer diagnosis, which is in agreement with previously reported data showing an association between poor survival with nulliparity that could be explained by the absence of breast tissue maturation that occurs during pregnancy and breastfeeding [22]. Moreover, Gleicher had hypothesized that the remaining stem cells in reproductive organs might be de-inhibited and lead to cancer development at advanced ages [36]. Further investigations are needed to better understand this relevant association.

In our cohort, treatment did not show significant difference, however we noted a higher proportion of treated young patients suggesting that young patients are more aggressively treated than older patients are. As already reported, tumors in young women were more aggressive. These finding are in total agreement with results obtained by El Saghir *et al*. [37].

In this study, 12.9% of young women and 17.47% of older women have developed metastasis. In the young group, metastasis appears to be correlated with positive progesterone expression and oral contraceptive use (*p* = 0.03). It is widely accepted that oral contraceptives are high risk factor of breast cancer. Moreover, the delay of diagnosis registered in the young group, may also explain the rapid progression of the disease [34, 35].

The main limitation of the study is the absence of date of death in the medical records, which limited the calculation of overall survival. Moreover, the lack of information regarding some parameters could have influenced their investigation.

## Conclusions

In Morocco, breast cancer is more frequent in young women as compared to western countries. Breast cancer in young women is more aggressive and is diagnosed late, leading to an intensive treatment. We can also assume that the main factors associated with breast cancer development in young women are hormonal and reproductive status. Larger multi-institutional studies, including evaluation of genetic biomarkers, are needed to confirm our results and explain the high prevalence of breast cancer in young women to improve breast cancer management in Morocco.

## Abbreviations

AJCC: American Joint Committee on Cancer
BC: Breast cancer
CI: Confidence interval
EFS: Event free survival
FISH: Fluorescent *in situ* hybridization
HR: Hazard ratio
IHC: Immunohistochemistry
LN: lymph nodes
PgR: Progesterone receptor
pTNM: pathological Tumor Node Metastasis
ER: Estrogen receptor
Her2: Human epidermal growth factor receptor 2
TNBC: triple negative breast cancer
NST: No Special Type
SBR: Scarff-Bloom Richardson classification
WHO: World Health Organization
